# Fast clustering and cell-type annotation of scATAC data using pre-trained embeddings

**DOI:** 10.1093/nargab/lqae073

**Published:** 2024-07-05

**Authors:** Nathan J LeRoy, Jason P Smith, Guangtao Zheng, Julia Rymuza, Erfaneh Gharavi, Donald E Brown, Aidong Zhang, Nathan C Sheffield

**Affiliations:** Center for Public Health Genomics, School of Medicine, University of Virginia, Charlottesville, VA 22908, USA; Department of Biomedical Engineering, School of Medicine, University of Virginia, Charlottesville, VA 22904, USA; Center for Public Health Genomics, School of Medicine, University of Virginia, Charlottesville, VA 22908, USA; Department of Biochemistry and Molecular Genetics, School of Medicine, University of Virginia, Charlottesville, VA 22908, USA; Child Health Research Center, School of Medicine, University of Virginia, Charlottesville, VA 22908, USA; Department of Computer Science, School of Engineering, University of Virginia, Charlottesville, VA 22908, USA; Center for Public Health Genomics, School of Medicine, University of Virginia, Charlottesville, VA 22908, USA; Center for Public Health Genomics, School of Medicine, University of Virginia, Charlottesville, VA 22908, USA; School of Data Science, University of Virginia, Charlottesville, VA 22904, USA; School of Data Science, University of Virginia, Charlottesville, VA 22904, USA; Department of Systems and Information Engineering, University of Virginia, Charlottesville, VA 22908, USA; Department of Biomedical Engineering, School of Medicine, University of Virginia, Charlottesville, VA 22904, USA; Department of Computer Science, School of Engineering, University of Virginia, Charlottesville, VA 22908, USA; School of Data Science, University of Virginia, Charlottesville, VA 22904, USA; Center for Public Health Genomics, School of Medicine, University of Virginia, Charlottesville, VA 22908, USA; Department of Biomedical Engineering, School of Medicine, University of Virginia, Charlottesville, VA 22904, USA; Department of Biochemistry and Molecular Genetics, School of Medicine, University of Virginia, Charlottesville, VA 22908, USA; Child Health Research Center, School of Medicine, University of Virginia, Charlottesville, VA 22908, USA; Department of Computer Science, School of Engineering, University of Virginia, Charlottesville, VA 22908, USA; School of Data Science, University of Virginia, Charlottesville, VA 22904, USA; Department of Public Health Sciences, School of Medicine, University of Virginia, Charlottesville, VA 22908, USA

## Abstract

Data from the single-cell assay for transposase-accessible chromatin using sequencing (scATAC-seq) are now widely available. One major computational challenge is dealing with high dimensionality and inherent sparsity, which is typically addressed by producing lower dimensional representations of single cells for downstream clustering tasks. Current approaches produce such individual cell embeddings directly through a one-step learning process. Here, we propose an alternative approach by building embedding models pre-trained on reference data. We argue that this provides a more flexible analysis workflow that also has computational performance advantages through transfer learning. We implemented our approach in scEmbed, an unsupervised machine-learning framework that learns low-dimensional embeddings of genomic regulatory regions to represent and analyze scATAC-seq data. scEmbed performs well in terms of clustering ability and has the key advantage of learning patterns of region co-occurrence that can be transferred to other, unseen datasets. Moreover, models pre-trained on reference data can be exploited to build fast and accurate cell-type annotation systems without the need for other data modalities. scEmbed is implemented in Python and it is available to download from GitHub. We also make our pre-trained models available on huggingface for public use. scEmbed is open source and available at https://github.com/databio/geniml. Pre-trained models from this work can be obtained on huggingface: https://huggingface.co/databio.

## Introduction

Data from the single-cell assay for transposase-accessible chromatin using sequencing (scATAC-seq) can interrogate complex regulatory networks at the single-cell level, elucidating the cellular mechanisms that drive cell-to-cell heterogeneity. The power of scATAC-seq data has motivated the development of new computational approaches for analysis of these data (1–12). Despite these advances, scATAC-seq analysis continues to face two key challenges: (i) high dimensionality and (ii) inherent sparsity of the data (13,14).

scATAC-seq analysis often includes two critical tasks: (i) dimensionality reduction followed by clustering and (ii) cell-type annotation of cell clusters. For the dimensionality reduction task, numerous methods have been developed, such as SCALE and scBasset, which use variational autoencoders and convolutional neural networks to learn low-dimensional representations of single cells for downstream clustering tasks (1,2). Other methods include ChromVAR, cisTopic, SnapATAC and ArchR, which leverage latent semantic indexing (LSI) and topic modeling to cluster individual cells (3–5,9). These methods usually require complex processing pipelines and large compute power. The second task, cell-type annotation, is less well served, with most current methods simply repurposing cell-type annotation tools from scRNA-seq (15). Methods developed for scATAC are few and suffer notable limitations. First, they mainly take a cross-modality approach, integrating data from reference scRNA-seq sets, so they are limited in relying on a secondary data modality. Finding an appropriate secondary dataset to complement the unlabeled set can be difficult (16). Second, many supervised methods require model training to predict cell types from a fixed output. This can make the discovery of novel cell types a challenge (12). Finally, these methods are notoriously compute-intensive (17), a limitation that has grown more problematic as atlas-level datasets have emerged.

Here, we address these challenges with an alternative approach to scATAC-seq dimensionality reduction and cell-type annotation using pre-trained embedding models. Our method improves both dimensionality reduction and cell-type annotation by significantly reducing the computational time and complexity of these workflows, with the added benefit of leveraging information from high-quality reference datasets. Instead of analyzing datasets end to end, we use unsupervised learning to model the patterns of regulatory region co-occurrence in reference datasets, and then transfer this knowledge to new, unseen data. While other transfer learning methods do exist, nearly all focus on the integration of scRNA-seq data with scATAC-seq (16,18,19). Moreover, methods that do focus on transfer learning for scATAC-seq data (*e.g*. 20) have limitations. No such method yet utilizes publicly available pre-trained models, has been used for cell-type annotation, or includes a software framework to facilitate easy analysis. Such a framework could drastically speed up analysis. To that end, we designed our method to remedy these shortcomings. We implemented this method in scEmbed, an unsupervised machine-learning method that learns low-dimensional representations of genomic regions from scATAC-seq datasets.

We first show that scEmbed performs well for dimensionality reduction and clustering while maintaining robustness to data loss. Moreover, by leveraging models pre-trained on reference data, scEmbed drastically reduces the time and complexity of scATAC-seq analysis. Finally, we build a cell-type annotation system by exploiting the learned embeddings produced by pre-trained embedding models without needing any external data modalities. Our system can accurately annotate unseen data in seconds using pre-trained reference models. scEmbed takes a new approach to scATAC-seq analysis by focusing on ATAC-seq data alone. scEmbed builds high-quality embeddings of genomic regions en route to single cells, which offers flexibility and speed for a wide range of downstream tasks.

## Materials and methods

### scEmbed architecture

**Figure 1.**
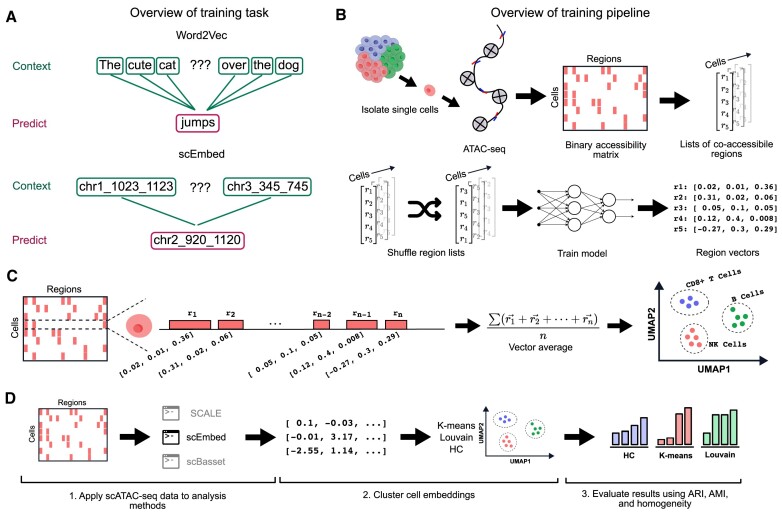
Overview of scEmbed architecture and benchmarking workflows. (**A**) scEmbed leverages Word2Vec as its model. Word2Vec learns to predict words given a semantic context. Similarly, scEmbed learns to predict genomic regions, given a genomic context. This is unsupervised, and uses the patterns of genomic region co-occurrence to learn representations of individual regions. (**B**) Overview of the scEmbed learning process, starting with scATAC-seq data. (**C**) Once region embeddings are learned, they can be used to construct cell embeddings by averaging the embeddings of regions accessible in each cell. We use cell embeddings for downstream tasks of clustering and cell-type prediction. (**D**) Diagram showing three steps of the benchmarking process.

scEmbed adapts our previous work, Region2Vec (21), to single cells. The model is a modified unsupervised word2vec (22) model that learns to predict genomic region co-accessibility (Figure 1A). Briefly, scEmbed treats each cell as a document and its accessible regions as words. We used the gensim implementation of Word2Vec as the core model for scEmbed. Word2Vec has many configurable hyperparameters (22), including context window size, embedding size, learning rate scheduling and number of epochs. All experiments were conducted with a fixed set of hyperparameters. We used defaults for scEmbed, informed by experiments on Region2Vec optimization (23). Specifically, we use a window size of 5 and an embedding dimension of 100. We also use 100 epochs for all experiments unless otherwise noted. We adopt an exponential learning rate schedule with a decay rate of 0.95.

Word2Vec takes lists of words as input. To this end, we designed a way to convert a binary accessibility matrix into list of words that are compatible with Word2Vec’s acceptable input corpus format. scEmbed expects binary accessibility matrices with cells as rows and regions as columns. Briefly, we treat each cell as a sentence and each co-accessible region in the cell as a word. Using this convention, we generate input for Word2Vec in three steps. First, we take an individual cell and identify each region where it shows signal. We define signal as anything greater than zero. Second, we take the corresponding region that shows signal and convert it into a word by concatenating the chromosome, start and end values with an underscore (chr_start_end). This process is completed for each region with signal in the cell, and a list of ‘words’ is constructed. Context is simulated by shuffling these regions in each document (Figure 1B). Shuffling is necessary since co-accessible regions have no inherent order, and Word2Vec learns by context. We repeat this process for each cell in the accessibility matrix. After training, cell embeddings are constructed by averaging region vectors for each cell, which are then used for tasks such as clustering, analysis, or transfer learning (Figure 1C).

### scEmbed model benchmarking and evaluation

To validate scEmbed, we followed an earlier approach (13) to benchmark it on clustering tasks using published reference scATAC data. We leveraged four main datasets in this work: 1) the Buenrostro2018 dataset, single-cell chromatin accessibility profiles from 10 human hematopoietic cell types (24); 2) Luecken2021, a multimodal single-cell benchmarking dataset of 120 000 single cells from the human bone marrow of 10 diverse donors measured with two commercially available multimodal technologies (25); 3) 10X genomics 5k PBMCs, a single-cell dataset of 5000 peripheral blood mononuclear cells from a healthy donor; and 4) a synthetic bone marrow dataset, a binary accessibility dataset described and provided by Chen *et al.* (13).

We benchmarked scEmbed on the Buenrostro2018 dataset (24) as well as a more recent and comprehensive scATAC-seq dataset from Luecken2021 (25) (Figure 1D). We trained scEmbed for 100 epochs (Supplementary Figure S1) then used the resulting region embeddings to construct cell embeddings. Following previous benchmarking procedures, we clustered the cell embeddings with three clustering methods: K-means, hierarchical clustering (HC), and Louvain clustering. There are two scenarios for which we can evaluate clustering: known ground-truth labels and unknown ground-truth labels. The synthetic bone marrow, Buenrostro2018, and Luecken2021 datasets have known ground-truth labels while the PBMC data have unknown ground-truth labels. When ground-truth labels are known, we employ three scores: the adjusted rand index (ARI), the adjusted mutual info score (AMI), and the homogeneity score. When ground-truth labels are not known, we use the Residual Average Gini Index (RAGI) (13).

### Dropout experiments

To explore its transfer learning ability and test robustness to missing data, following Xiong *et al.* (1), we evaluated scEmbed on datasets with increasing levels of information loss. Starting from the already sparse Buenrostro2018 cell-feature matrix (2.8% non-zero) (26), we randomly dropped non-zero values in the binary accessibility matrix until ∼80% of the non-zero data were lost, resulting in a matrix that was 0.5% non-zero (Supplementary methods; Supplementary Figure S3).

### scEmbed transfer learning and projection

**Figure 2.**
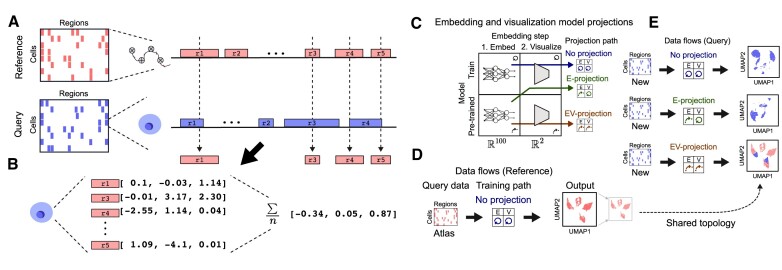
scEmbed projection workflows. (**A**) Diagram of overlap analysis depicting how a new cell from a new dataset (blue) is cast in the feature space of the data used for the pre-trained model (red). (**B**) Diagram showing the computation of embeddings for new, unseen data. This is achieved using average pooling of region embeddings. (**C**) Diagram showing scEmbed’s three projection paths. (**D**) Overview of the standard ‘no-projection’ data flow. (**E**) Overview of three data flows for new data. EV-projection places the new data in the same latent space as the reference data.

A key innovation in scEmbed is that it uses a two-step training process, rather than the common single-step approach. In the first step, scEmbed learns embeddings of genomic regions rather than cells. In the second step, the region embeddings are used to build cell embeddings. An advantage of this two-step approach is that the region embeddings can be used to build cell embeddings for new datasets. This transfer approach allows scEmbed to take advantage of pre-trained reference models. We call this ‘projection’ because we ‘project’ new data into the latent space of the original dataset, creating cell embeddings for new data using a pre-trained model. Projection occurs in three steps: first, we train a model on reference data to produce region embeddings for each region in the reference consensus region set. For datasets where consensus peaks do not exist, methods that create such sets from raw data could be used as a pre-processing step (26). Second, we take a new single-cell dataset and map the regions to the reference consensus region set using region overlaps (Figure 2A; Supplementary methods). This represents each single cell in the new dataset using the set of regions from the reference dataset, for which we also have region embeddings from the reference model. Finally, we compute the average of all region embeddings for each cell in the new dataset (Figure 2B). This approach leverages the information from a larger atlas of accessibility data to analyze a new dataset. In fact, the original training data need not come from scATAC-seq at all. Using this approach, a model trained with bulk ATAC-seq could be used to project scATAC-seq data. This provides an enormous advantage by utilizing the patterns of region co-occurrence from the vast volume of publicly available region set data to inform cell embeddings of single-cell data.

### Projection visualization and cell-type annotation

Finally, we sought a way to visualize projected cells. We reasoned that this approach could annotate clusters for projected cells, allowing us to borrow annotation information from the reference model. To build such a system, we distinguish between three data flows that can occur with scEmbed: no projection, E-projection and EV-projection (Figure 2C). First, we train a reference model using the typical no projection flow (Figure 2D). This is the standard workflow of training a new scEmbed model on some input data and visualizing the resulting embeddings by fitting a UMAP model to reduce the dimensionality to two. Then, given a new query dataset, we could analyze it with any of the three data flows (Figure 2E). In a no projection flow, we would not use the reference model at all; we train and visualize using only the new dataset. In embedding-only projection data flow (E-projection), new data are first embedded using the pre-trained reference model. These embeddings may then be visualized by fitting a UMAP model to reduce dimensionality to two dimensions. The third data flow, and the novel innovation that accomplishes our goal of reference-based visualization, is the embedding and visualization workflow (EV-projection). In this data flow, new data are first embedded using the pre-trained reference model, as in E-projection; then, these embeddings are further projected through a UMAP model that was fit on the reference data embeddings, rather than newly fit. With the EV-projection flow, plotting the two-dimensional cell representations on top of the reference data UMAP plot allows one to visualize where in the original embedding space the new data ended up. This is possible because the EV-projection re-uses the same topology from the UMAP model fit to the reference data (Figure 2D, E).

## Results

### scEmbed is competitive with existing scATAC-seq methods

**Figure 3.**
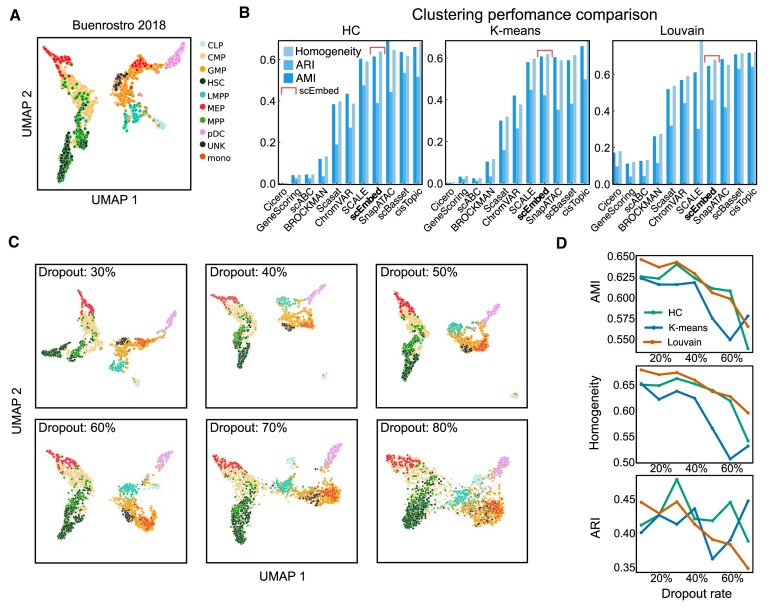
scEmbed benchmarks competitively with existing approaches. (**A**) UMAP of scEmbed cell embeddings for the Buenrostro2018 dataset. (**B**) Benchmark results of three clustering methods: hierarchical clustering (HC), K-means and Louvain. Results were evaluated using three metrics: adjusted mutual information (AMI), adjusted rand index (ARI) and homogeneity. (**C**) UMAP plots showing clusters of scEmbed cell embeddings following data loss. (**D**) Line plots showing the change in three clustering metrics (ARI, AMI and homogeneity) as a function of dropout rate. scEmbed accurately clusters single cells up to nearly 80% data loss.

When benchmarked against Buenrostro2018 (24), scEmbed clusters cells of the same type visually (Figure 3A). scEmbed performed similarly to the best-performing scATAC-seq methods, including SCALE, scBasset, cisTopic and SnapATAC (Figure 3B). This performance was achieved with minimal pre-processing of the data and a completely unsupervised learning workflow. In addition to the Buenrostro2018 dataset, we also benchmarked scEmbed on another, more recent and comprehensive scATAC-seq dataset from Luecken *et al.* (25). Again, comparing clusters with ground truth labels, scEmbed performs well (Supplementary Figure S2).

### scEmbed is robust to data loss

For the dropout evaluation, even at a dropout rate of 80%, scEmbed was able to visually cluster cells of the same type (Figure 3C). Evaluation metrics show that scEmbed retained clustering accuracy even when faced with substantial data loss (Figure 3D). This is comparable with other scATAC clustering methods which are robust to data loss (13). These findings confirm that scEmbed can learn rich biological knowledge, even for the most sparse datasets.

### Projected cell embeddings cluster cells accurately using pre-trained models

An important feature of scEmbed is that it can cluster unseen data without training a new model using projection (see the Materials and methods). We next sought to assess this projection process by asking whether scEmbed could cluster a new dataset based entirely on a pre-trained model. First, we trained a model on the original Buenrostro2018 dataset (24); second, we took a new dataset, 10X genomics 5k PBMCs from a healthy donor, and projected each cell into the original space. We used these single-cell embeddings directly for UMAP visualization and clustering analysis. To assess the quality of the projection, we assumed that eight distinct cell populations existed (13) and took advantage of marker gene analysis to assign labels to each cell. We use the RAGI score to evaluate the clustering ability of scEmbed (13) (Supplementary methods).

**Figure 4.**
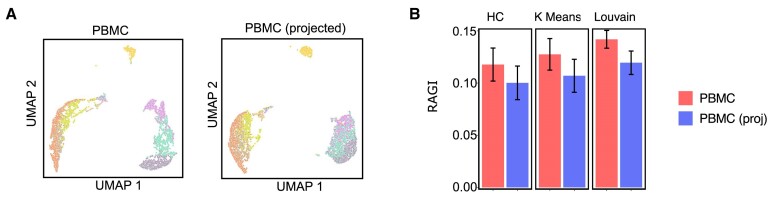
Projection enables robust clustering of single cells for unseen data. (**A**) UMAP plots of a single dataset when used to train a model (left) or when simply projected through a pre-trained model (right). The cells cluster similarly. (**B**) RAGI score plots for the original dataset embeddings and projected cell embeddings for each clustering method: hierarchical clustering, K-means and Louvain. RAGI scores are based on a sampling strategy and computed as the mean RAGI score across all samples. Error bars indicate one standard deviation away from the mean.

We found that the projected cell analysis showed no marked differences in clustering proficiency when compared with the embeddings produced by conventional model training. The UMAP plots were visually similar (Figure 4A), indicating similar clustering performance. To further explore the difference, we next evaluated clustering performance using a repeated subsampling strategy (Supplementary Figure S4) that consisted of four steps: (i) train a new model on the PBMC data alone; (ii) repeatedly subsample 1000 cells and compute their embeddings using the new model and the Buenrostro2018 model using projection; (iii) cluster the cells using three strategies (HC, K-means and Louvain); and (iv) compute the RAGI score with these 1000 subsampled cells. The scores were then averaged across all subsamples. Our results showed that the RAGI score between the original and projected datasets did not differ significantly, indicating similar clustering performance (Figure 4B).

### Pre-trained models from reference datasets can be used to annotate cell clusters

**Figure 5.**
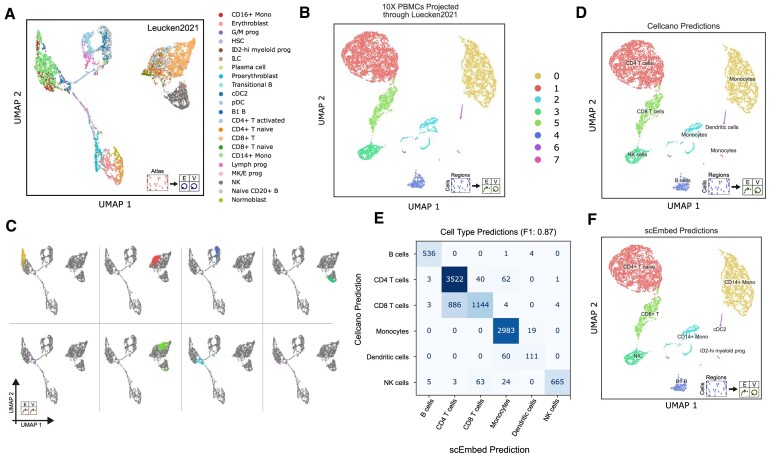
Pre-trained embedding models can be exploited for cell-type annotation tasks. (**A**) UMAP plot of the Luecken2021 dataset used to train the reference model. (**B**) UMAP of embeddings produced for the new PBMC dataset through E-projection. (**C**) Plots showing the EV-projection data flow applied to the new PBMC dataset. Gray cells represent the reference topology; colored cells are projected new PBMC data. Separate plots depict individual clusters for visual clarity. (**D**) UMAP plots showing the cell labels assigned by Cellcano. (**E**) Confusion matrix of scEmbed classification results compared with Cellcano. (**F**) UMAP plot showing cell type labels assigned by scEmbed.

Convinced that pre-trained models could be used to visualize an unseen query dataset, we next asked whether this approach could be used to annotate cell types without training a model. We first built a reference model using the Luecken2021 multiomic dataset (25), a first-of-its-kind multimodal benchmark dataset of 120 000 single cells from the human bone marrow of 10 diverse donors measured with two commercially available multimodal technologies. Using scEmbed and the ‘no projection’ data flow, we trained a model and clustered the resulting embeddings (Figure 5A; Supplementary Figure S5). This model served as the reference for all downstream experiments with a new PBMC dataset from 10X genomics. Using E-projection, scEmbed creates visually distinct clusters of single cells (Figure 5B). To visualize these embeddings in the context of the original embedding topology, we employ EV-projection. Each identified cluster from E-projection aggregates to a distinct location in the original UMAP embedding topology of the Luecken2021 model (Figure 5C).

Confident our pre-trained Luecken2021 embedding model was distinctly clustering the new PBMC dataset, we sought to assign cell-type labels to each cluster. We used Cellcano, a new scATAC-seq cell annotation method, to assign ground-truth labels to each cluster of the E-projected PBMC embeddings for evaluation of our method (12) (Figure 5D). Our cell-type annotation system was limited by the cell types annotated in Luecken2021; as such, we mapped each scEmbed prediction class to a corresponding Cellcano class for comparison (Supplementary Table S1) after following their annotation procedure (see the Materials and methods; Supplementary Figure S6). Using a simple k-nearest-neighbor (KNN) classification algorithm (see the Supplementary methods), scEmbed was highly consistent with the Cellcano labels (F1 = 0.87, Figure 5E). However, without class mapping, scEmbed offers higher specificity of cluster identity and even identifies a cluster of ID2-hi myeloid progenitor cells not found with Cellcano (Figure 5F). Moreover, this workflow enables researchers to quickly try new models trained on many different cell types and rapidly discover cell types in their data. Using our projection system, researchers can avoid training a new model each time they want to use a new reference dataset, which is a common approach in many modern cell-type annotation systems (7,12,27). The entire process of dimensionality reduction, clustering and annotation took <10 min on a laptop, and we observed similar time savings across several models (Supplementary Figure S7). Thus, we conclude that EV-projection is a promising approach for fast visualization and annotation of new data.

## Discussion

In this work, we demonstrate the robustness and versatility of scEmbed, a new tool for the analysis of scATAC-seq data. scEmbed differs from existing methods in that instead of learning embeddings of individual cells directly, it first learns embeddings of genomic regulatory regions and then uses these to compute cell embeddings. We demonstrate how this approach allows scEmbed to use pre-trained genomic region embedding models to effectively cluster data not seen by the model. Our evaluation of scEmbed against existing scATAC-seq methodologies demonstrates its efficacy and competitiveness, even with a relatively simple network architecture. scEmbed performs well, even when faced with severe data loss. The standout feature of scEmbed is its capability to repurpose learned region embeddings for downstream analysis tasks. This approach provides flexibility and efficiency, setting it apart from other currently available tools.

By exploiting region overlaps and applying previously learned region embeddings, we have formulated a novel method for representing unseen scATAC-seq data within the latent space of the original training data. This process, termed ‘projection’, yielded superb clustering of cells, showing no significant decrease in performance compared with models trained entirely on the new dataset. This performance underscores the potential of scEmbed in the context of ATAC-seq transfer learning tasks and opens up exciting possibilities for future research. Moreover, we emphasize the novelty of scEmbed in its ability to engage in transfer learning without the need for another data modality like scRNA-seq, which is overwhelmingly required by current methods. Future studies will explore the ability of our model to learn and extract overarching regulatory patterns from publicly available data. This learning, coupled with the inherent transferability of scEmbed, will empower researchers to fine-tune the models for specific downstream tasks, enabling gains in performance, efficiency, and flexibility.

Finally, we leveraged embeddings computed by scEmbed and its pre-trained models to build a novel cell-type annotation system. Our method is consistent with current scATAC-seq cell-type annotation implementations, with the added advantage of requiring no external data modalities. Furthermore, by exploiting pre-trained models and pre-computed cell embeddings from reference datasets, the scEmbed annotation system can easily scale to millions of cells and uses only a fraction of the compute time. This is because utilization of a pre-trained model requires only interval overlap analysis to map the new data into the feature space on which the model was trained (Supplementary Figure S7). We have made the pre-trained models used in this study available for download and use on huggingface. To facilitate model sharing and usability even further (28), we have built software packages to easily download and use these models within Python. Moreover, these same packages can be used to train new models or fine-tune public ones on custom datasets. We hope that these resources will enable researchers to leverage the power of scEmbed for their own research.

The integration of unsupervised learning with transfer learning may offer new directions for other bioinformatics tasks that are similarly burdened by the challenges of high dimensionality and data sparsity. Furthermore, the deployment of pre-trained models for reference datasets may inspire novel methodologies for efficient and accurate cell-type annotation systems across different data modalities. In the future, the pre-training approach of scEmbed could be adapted for use with cross-modality methods that span data types (29). In conclusion, scEmbed’s ability to distill meaningful representations from vast, complex scATAC-seq datasets, and repurpose this knowledge for rapid and accurate analysis of new datasets, has great potential. This work is a step towards developing more efficient, scalable, and flexible tools for genomic data analysis. The opportunities unlocked by scEmbed for research and clinical application promise exciting advancements in the comprehension of cellular heterogeneity and the intricate regulatory networks that drive it.

## Supplementary Material

lqae073_Supplemental_File

## Data Availability

scEmbed is open source and available at https://github.com/databio/geniml and https://doi.org/10.5281/zenodo.11642482. Pre-trained models can be obtained on huggingface: https://huggingface.co/databio.
