# Supplementary material for: Fast clustering and cell-type annotation of scATAC data using pre-trained embeddings
Source: NAR Genom Bioinform. 2024 Jul 5;6(3):lqae073. doi: 10.1093/nargab/lqae073 (PMC11224678; doi:10.1093/nargab/lqae073)
Supplement: lqae073_Supplemental_File [file lqae073_supplemental_file.pdf]

Supplemental methods

Data and data processing

Detailed overview of datasets

Luecken2021. The Luecken2021 dataset is a multimodal single-cell benchmarking dataset (27). The data is a first-of-its-kind multimodal benchmark dataset of 120,000 single cells from the human bone marrow of 10 diverse donors measured with two commercially-available multi-modal technologies: nuclear GEX with joint ATAC, and cellular GEX with joint ADT profiles. The data was retrieved from the gene expression omnibus (GEO) using the GEO accession GSE194122.

Buenrostro2018. The Buenrostro2018 dataset consists of single-cell chromatin accessibility profiles across 10 populations of immunophenotypically defined human hematopoietic cell types (26). Deduplicated single-cell bam files along with a consensus peak set were provided by Chen *et. al.* (13). Using `bedtools` (32), region overlaps with the consensus peak set were computed for each bam file at a minimum overlap of 1bp. Using the `-c` flag, the number of overlaps with each region in the consensus peak set was calculated. Overlap count files were subsequently converted into a cell by peak binary accessibility matrix formatted as a comma-separated-value file (`csv`). Finally, the binary accessibility `csv` was converted into a `scanpy` `AnnData` object using the `scanpy.read_csv` API. This was used as input to the scEmbed model.

5k PBMC. The PBMC dataset comes from 10X genomics and consists of peripheral blood mononuclear cells (PBMCs) from a healthy donor. Three files were downloaded directly from the 10X genomics website: 1) the sparse peak matrix in `.mtx` format, 2) the cell barcode labels in `tsv` format, and 3) the consensus peak set in `bed` format. Using Python, along with `pandas` and `scanpy`, these files were processed into a `scanpy` `AnnData` object. This was used as input to the scEmbed model.

Synthetic Bone Marrow. The synthetic bone marrow dataset was described and provided by *Chen et. al.*(13). The binary accessibility matrix was downloaded directly from the Pinello Lab's GitHub as a `.rds` file. Using R, this file was read, parsed, and exported as a `csv`. Like the previous two datasets, this `csv` was processed into a `scanpy` `AnnData` object using `pandas` and `scanpy`.

Clustering

We use three clustering algorithms: Hierarchical clustering (HC), k-means clustering, and Louvain clustering. For HC and k-means, we use the `scikit-learn` implementations. When ground-truth labels were known for a particular dataset, we used the number of unique labels to set the number of clusters to generate. Otherwise, we used prior knowledge to estimate the number of unique cell populations we would expect to find. For Louvain clustering, we use the `scanpy` implementation. Louvain is agnostic to a specified number of clusters. As such, we iteratively applied clustering to datasets while slowly increasing the resolution value from 0 to 3. With each iteration, the number of clusters was stored in a list along with the corresponding resolution. Once complete, we employed binary search on the list to identify the resolution that gave us the desired number of clusters. This value was used to generate the final clustering solution.

Visualization

We used uniform manifold approximation and projection (UMAP) to visualize single-cell embeddings (33). We used the `umap-learn` Python package and specified two dimensions for each visualization. In addition, a random state of 42 was set for visualization workflows. All other parameters were set to package defaults.

Clustering evaluation

Evaluation metric

Three scores are employed when a dataset has ground-truth labels: The adjusted rand index (ARI), the adjusted mutual info score (AMI), and the homogeneity score. When ground-truth labels are not known, we use the Residual Average Gini Index (RAGI) (13). We describe them in detail below.

Adjusted Rand Index

The ARI is a metric for evaluating the similarity between two data clusterings. This is achieved by counting pairs that are assigned to the same cluster label. Mathematically, it is computed by:

$$ARI = \frac{\sum_{ij}\binom{n_{ij}}{2} - [\sum_i \binom{a_i}{2}\sum_j \binom{b_j}{2}]/\binom{n}{2}}{\frac{1}{2}[\sum_i \binom{a_i}{2} + \sum_j \binom{b_j}{2}] - [\sum_i \binom{a_i}{2}\sum_j \binom{b_j}{2}]/\binom{n}{2}}$$

where n_{ij}, a_i, b_j are diagonal values, row sums, and column sums respectively from the contingency table that describes the frequency distribution of the cluster labels from ground-truth and predicted clusterings.

We use the `adjusted_rand_score` function from the `scikit-learn` python package.

Adjusted Mutual Info Score

The AMI, intuitively, is a measure of the amount of information that two clusterings share. It's used to evaluate how well two clusterings agree with each other (34). We compute AMI through the `scikit-learn` package using the `adjusted_mutual_info_score` function.

Homogeneity Score

The homogeneity score is an entropy-based external cluster evaluation metric that measures how far from perfect an incorrect clustering solution is (35). We employ the `scikit-learn` `homogeneity_score` function to measure this metric for each dataset.

Residual Average Gini Index

When ground truth labels are unknown, all aforementioned evaluation metrics are no longer applicable. As such, we need a measure that can still evaluate dataset clustering based on what one would expect, given some sort of prior knowledge about the system. For this, we employ a similar strategy described by *Chen et. al.* called the Residual Average Gini Index (RAGI). Briefly, the RAGI score compares the accessibility of housekeeping genes with previously characterized marker genes (36). RAGI measures the average residual specificity of a clustering solution with respect to marker genes, suggesting that a good clustering solution should have clusters enriched for different marker genes and these genes should be highly accessible in only a few clusters, compared to the less informative housekeeping genes.

Data corruption and dropout experiments

Following a similar approach described by *Xiong et. al.* to evaluate SCALE (1), we randomly dropped out non-zero values at increments of 10% from 0.1 to 0.8. This resulted in increasingly more sparse feature matrices (Fig. S3). These matrices have been made more sparse than the original, already-sparse scATAC binary accessibility matrix. Using `choice` from the `numpy` python library, we changed all non-zero values in the feature matrix to 0 with a probability of Dropout Rate. The resulting matrix was saved and used for downstream analysis.

Transfer learning and data projection

E-projection

The E-projection method enables the transfer of learned knowledge from a pre-trained model to unseen datasets. It translates a new binary accessibility matrix into the space of the original dataset. For each cell in the new dataset, this projection occurs in three stages:

Region Overlap Computation. We first calculate region overlaps between the accessible regions in the new cell and the consensus peak set from the original dataset. This is achieved using interval trees, a data structure that facilitates efficient discovery of all intervals that overlap with any given interval or point. We use the `intervallist` package in python for these computations. To increase speed and performance for on-disk datasets, the augmented interval list (AIList) data structure could be used (37). Regions with signal that overlap are mapped onto the original dataset's consensus peak set.

Word-Key Conversion. Subsequently, each of the overlapping regions is transformed into its corresponding word-key. This is accomplished by joining the values of the chromosome, start, and end with an underscore (`chr_start_end`).

Embedding Transformation and Averaging. Finally, these word-keys are translated into their corresponding embeddings. Region embeddings are obtained by passing the regions corresponding one-hot vector through a single embedding layer. We denote a one-hot encoded vector for a given region as O_r, where r represents the specific region. We then denote the embedding weight matrix as W_e, where each column corresponds to the embedding of a particular region in embedding space. Given these symbols, the operation to translate a one-hot encoded vector into its corresponding embedding can be represented as: $E_r = W_e \cdot O_r$. An average is then calculated across these embeddings to derive a whole-cell embedding E_{cell}.

Formally, let C denote a single cell from the new dataset, R the original dataset's consensus peak set, and O the set of overlapping regions between C and R. For each region $r_i \in O$, we compute the embedding $E_{r_i} = g(r_i)$, where g is the embedding transformation $W_e \cdot O_{r_i}$. The whole-cell embedding is then computed by averaging these region embeddings:

$$E_{\text{cell}} = \frac{1}{n} \sum_{I=1}^{n} E_{r_i}$$

where n is the number of regions in O. These vector averages are computed with the `numpy` package in python.

EV-Projection

The EV-Projection procedure extends the E-Projection method by incorporating an additional visualization step. We begin by calculating region overlaps and converting regions to their corresponding embeddings. We then average these embeddings to compute a whole-cell embedding for each cell in the new dataset.

After obtaining the whole-cell embeddings E_{cell} from E-Projection, these embeddings are further transformed using the UMAP model fitted to the original data embeddings. The explicit equation for UMAP's transformation process on a new point is not straightforward to express in a simple mathematical formula due to the complexity of the algorithm (33). To that end, we denote this transformation function as f_{UMAP}. The EV-projection cell-embeddings as follows:

$$EV_{\text{cell}} = f_{\text{UMAP}}(E_{\text{cell}})$$

This transformation facilitates the visualization of the new data in the same low-dimensional space as the original data, providing a spatial relationship between new and original cells. This step aids in interpreting and comparing the accessibility landscape of the new cells in the context of the original cells.

Cell type classification

Embeddings of the Luecken2021 dataset

To classify new, unseen single cells we first generate an embedding for every cell in the Luecken2021 dataset (27). Each cell in the dataset, denoted as x_i, is transformed into a high-dimensional representation, or an embedding, denoted as e_i using the pre-trained model. Each embedding has an assigned ground-truth cell type label. The embeddings are generated with the no-projection procedure and stored alongside their metadata in a Qdrant database. Qdrant is open sourced and can be found on GitHub: https://github.com/qdrant/qdrant. Qdrant allows fast, convenient approximate nearest neighbor computation. We denote the set of all embeddings as $E = e_1, e_2, ..., e_n$, where n is the total number of cells in the Luecken2021 dataset. The corresponding cell type labels are represented as $L = l_1, l_2, ..., l_n$.

Approximate K-nearest-neighbor calculation

Given an unseen data point, x_u, the goal is to assign it a label by using the embeddings of the pre-labeled dataset. The first step towards this is to compute an embedding for the unseen data point, through E-projection. Next, we compute the approximate nearest neighbors to e_u using navigable small world graphs with controllable hierarchy (Hierarchical NSW, HNSW) (38). This is implemented in Qdrant with cosine distance and we simply query the database with the new embedding e_u.

Label transfer

Once the approximate k-nearest-neighbors are retrieved, we assign a label to e_u by performing a consensus vote among its k nearest neighbors. We denote the indices of these k nearest neighbors as $I = i_1, i_2, ..., i_k$. We then assign a label, l_u, to the unseen data point, x_u, based on the most common label among its k nearest neighbors, which can be formalized as:

$$l_u = \arg\max_{l \in L} \sum_{i=1}^{k} [l_{i_i} = l]$$

where $[l_{i_i} = l]$ is the Iverson bracket notation that equals 1 if the condition inside the brackets is met, and equals 0 otherwise. We use the Python `collections.Counter` object from the standard library to perform these computations.

Cluster annotation

After performing the label transfer procedure, the newly clustered dataset, which we'll denote as $C = C_1, C_2, ..., C_m$, where m is the total number of clusters and each cluster C_j is a set of data points, undergoes a final round of consensus voting for label assignment.

For each cluster C_j, we count the frequency of each label amongst all the data points within the cluster. Let $L_{C_j} = l_{j1}, l_{j2}, ..., l_{jn_j}$ represent the labels of data points within cluster C_j, where n_j is the total number of data points in C_j.

The label of cluster C_j, denoted as L_{C_j}, is then assigned based on the most frequent label among all data points in C_j. This can be formally defined as:

$$L_{C_j} = \arg\max_{l \in L} \sum_{i=1}^{n_j} [l_{ji} = l]$$

where $[l_{ji} = l]$ is the Iverson bracket notation that equals 1 if the condition inside the brackets is met, and equals 0 otherwise.

In this way, each cluster is assigned the label that is most represented among its constituent data points. The quality of cluster labeling is highly dependent on the accuracy of the initial label transfer step.

Evaluation of scEmbed cell type annotation

We use Cellcano, a novel scATAC-seq cell annotation method, to assign ground truth labels to our new PBMC data (12). We follow the online tutorials (https://marvinquiet.github.io/Cellcano/) and leverage their provided reference dataset to process the new PBMC data. Once ground truth labels have been assigned by Cellcano and putative cell types are assigned from scEmbed, we can compute the F1 score to measure the accuracy of our classification.

The F1 score is the harmonic mean of precision and recall, and provides a balance between these two measures. Precision is the number of true positives divided by the sum of true positives and false positives, and recall is the number of true positives divided by the sum of true positives and false negatives. Formally, these are defined as:

$$P = \frac{TP}{TP + FP}$$

$$R = \frac{TP}{TP + FN}$$

The F1 score is defined as:

$$F1 = 2 \times \frac{P \times R}{P + R}$$

To compute these measures, we compare the predicted labels, denoted as $L_p = l_{p1}, l_{p2}, ..., l_{pn}$, to the ground truth labels, denoted as $L_g = l_{g1}, l_{g2}, ..., l_{gn}$, where n is the total number of data points (or clusters). We utilize the `metrics.f1_score` function from `scikit-learn` to compute this value.

Supplemental references

32. Quinlan,A.R. and Hall,I.M. (2010) BEDTools: A flexible suite of utilities for comparing genomic features. *Bioinformatics (Oxford, England)*, **26**, 841–842.

33. McInnes,L., Healy,J. and Melville,J. (2020) UMAP: Uniform Manifold Approximation and Projection for Dimension Reduction. 10.48550/arXiv.1802.03426.

34. Vinh,N.X., Epps,J. and Bailey,J. (2009) Information theoretic measures for clusterings comparison: Is a correction for chance necessary? In *Proceedings of the 26th Annual International Conference on Machine Learning*, ICML '09. Association for Computing Machinery, New York, NY, USA, pp. 1073–1080.

35. Rosenberg,A. and Hirschberg,J. (2007) V-Measure: A Conditional Entropy-Based External Cluster Evaluation Measure. In *Proceedings of the 2007 Joint Conference on Empirical Methods in Natural Language Processing and Computational Natural Language Learning (EMNLP-CoNLL)*. Association for Computational Linguistics, Prague, Czech Republic, pp. 410–420.

36. Pliner,H.A., Shendure,J. and Trapnell,C. (2019) Supervised classification enables rapid annotation of cell atlases. *Nature Methods*, **16**, 983–986.

37. Feng,J., Ratan,A. and Sheffield,N.C. (2019) Augmented Interval List: A novel data structure for efficient genomic interval search. *Bioinformatics*, **35**, 4907–4911.

38. Malkov,Y.A. and Yashunin,D.A. (2016) Efficient and robust approximate nearest neighbor search using Hierarchical Navigable Small World graphs. *arXiv.org*.

Supplementary figures

Supplementary Table S1. *Label mapping between scEmbed and cellcano for consistent comparison of classification performance.*

scEmbed label	Cellcano label
B1 B	B cells
CD4+ T activated	CD4 T cells
CD4+ T naive	CD4 T cells
CD8+ T	CD8 T cells
CD8+ T naive	CD8 T cells
CD14+ Mono	Monocytes
cDC2	Dendritic cells
NK	NK cells
Naive CD20+ B	B cells

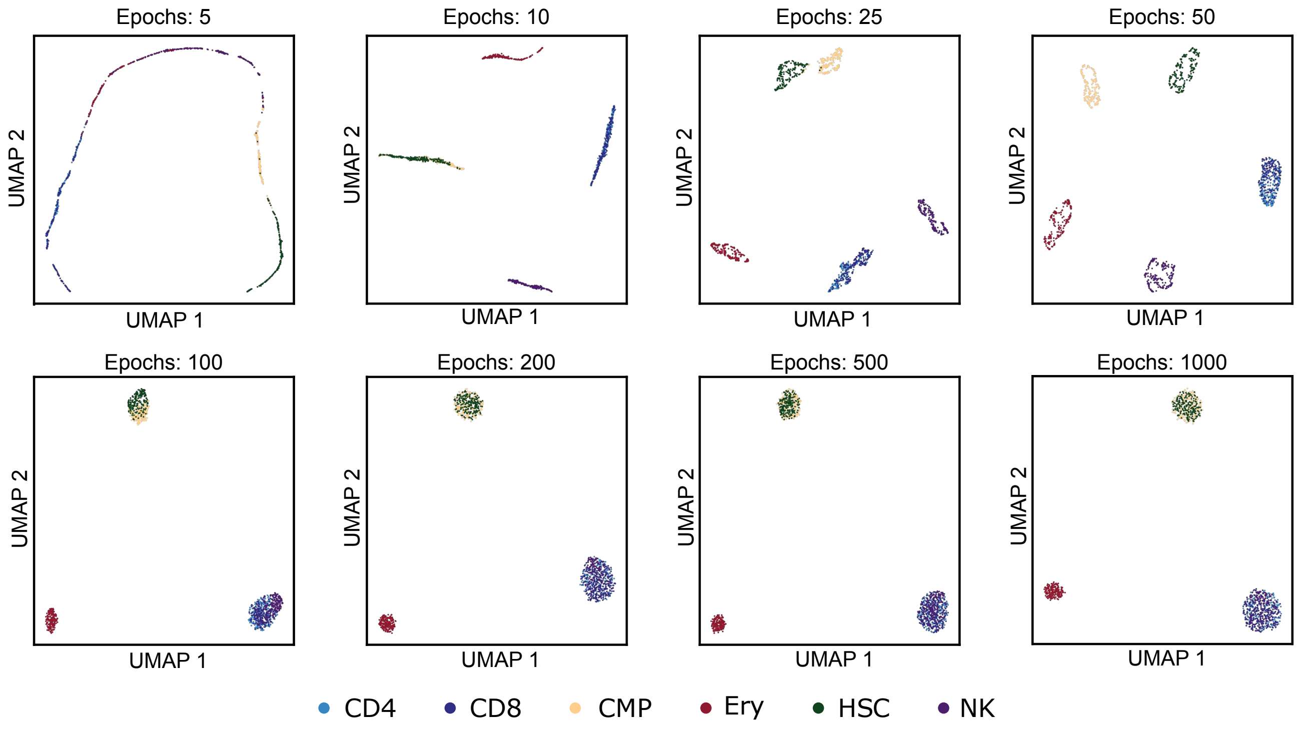

Supplementary Figure S1. Epoch tests show that scEmbed learns well after 100 epochs. *UMAP plots were generated after repeated model training using 8 different numbers of epochs for training. The model was trained on a synthetic bone marrow dataset described by Chen* et al. *UMAPs showed little change after 100 epochs.*

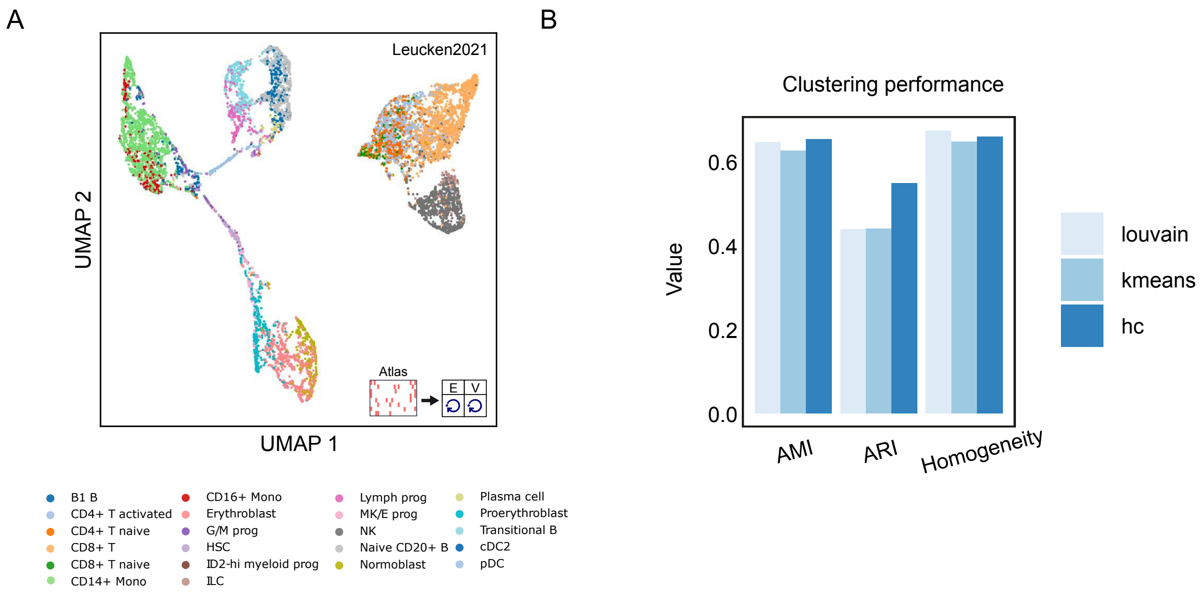

Supplementary Figure S2. scEmbed produces visually distinct clusters of the Luecken2021 dataset. A. *UMAP plot of the Luecken2021 dataset.* ***B.*** *ARI, AMI, and homogeneity scores for clusters produced by scEmbed using cell-type labels as the ground-truth.*

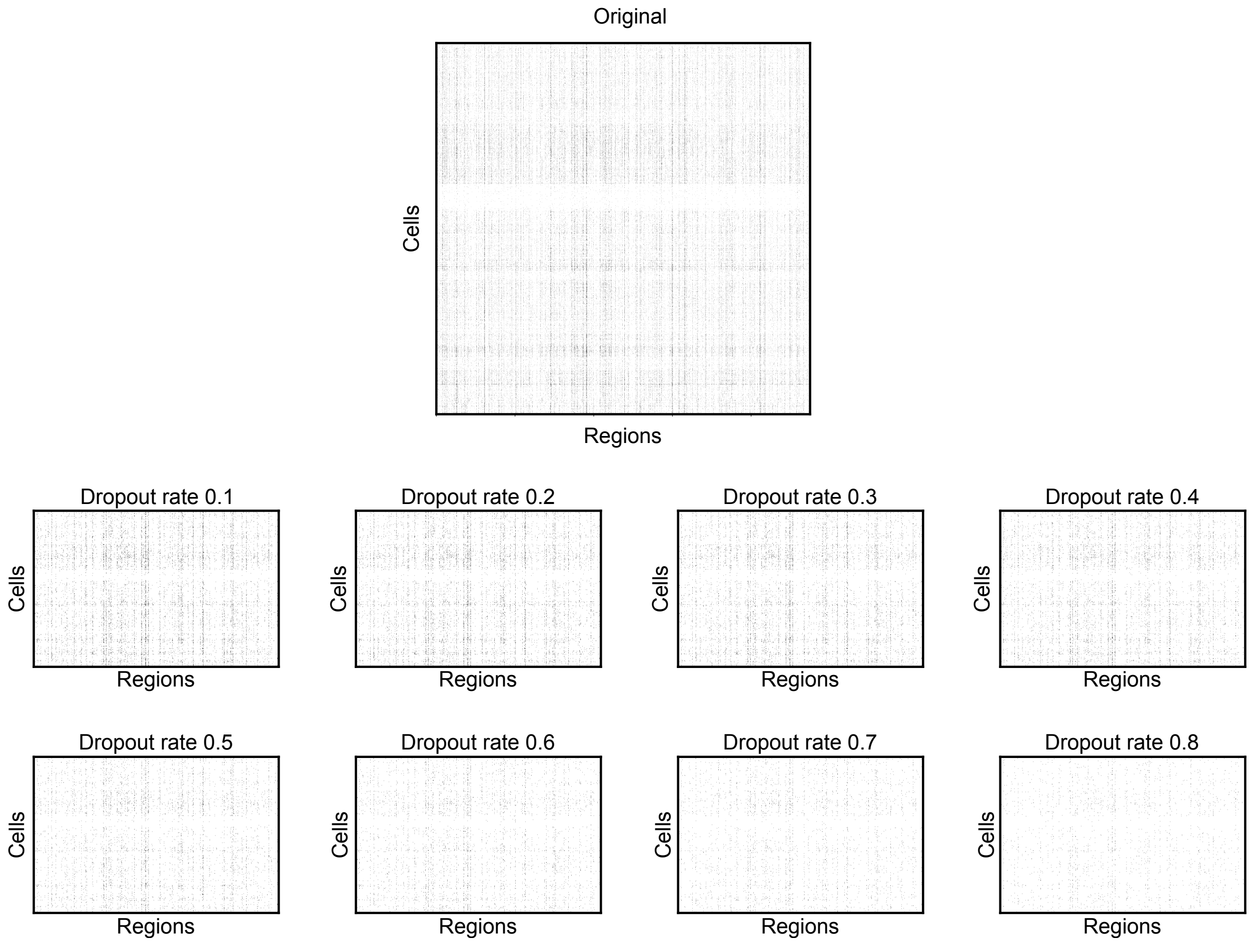

Supplementary Figure S3. Sparsity plots enable visualization of iterative data dropout. *Sparsity plots of the original matrix along with the matrix dropouts. Non-zero values decrease as the dropout rate increases from 10% to 80%.*

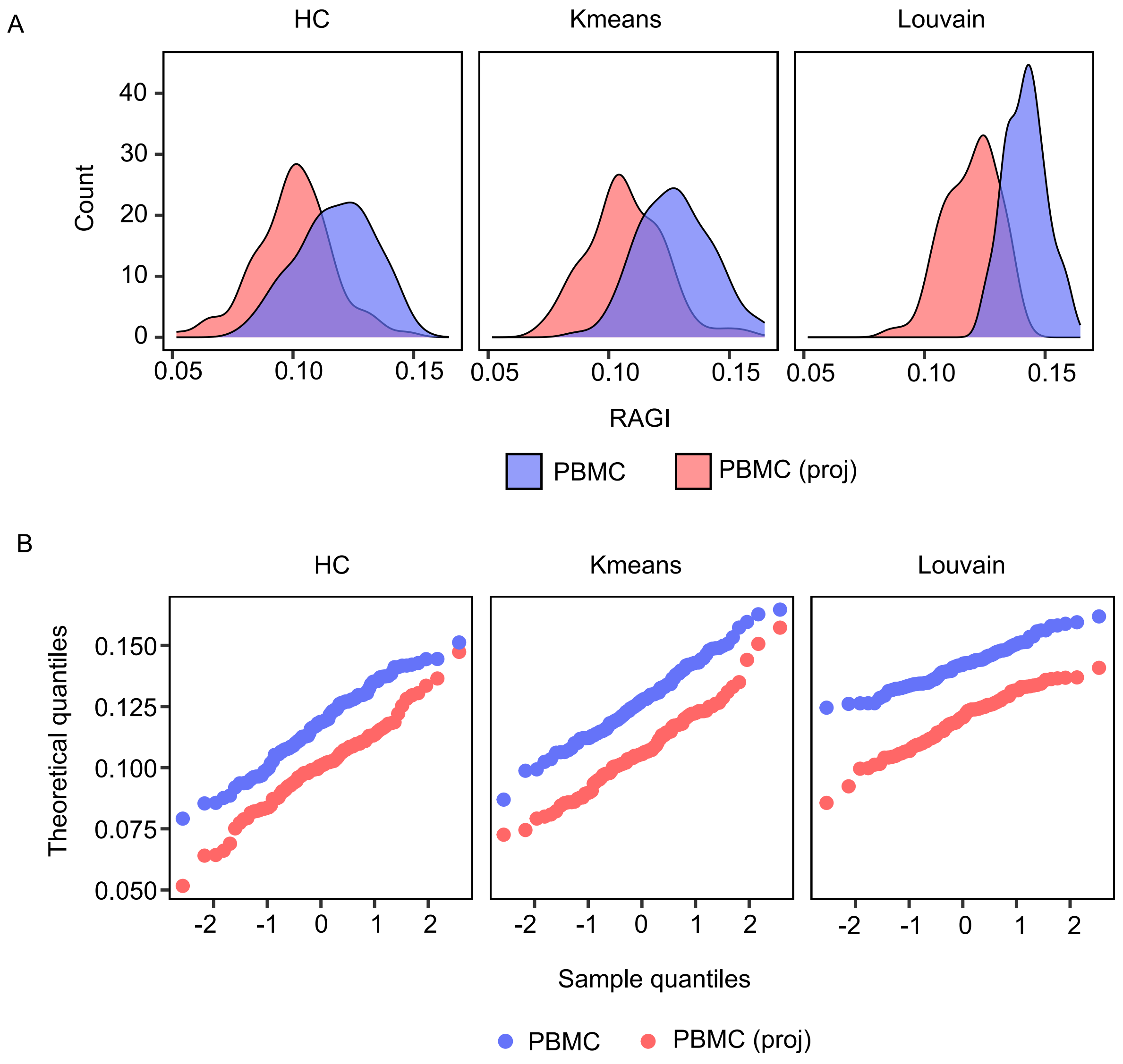

Supplementary Figure S4. Distributions of the RAGI scores for all subsampled cells. A. *Distribution of RAGI scores for cells with embeddings from the new model versus projection through the model trained on the Buenrostro2018 dataset.* ***B.*** *QQ plots of the RAGI scores for cells with embeddings from the new model versus projection through the model trained on the Buenrostro2018 dataset.*

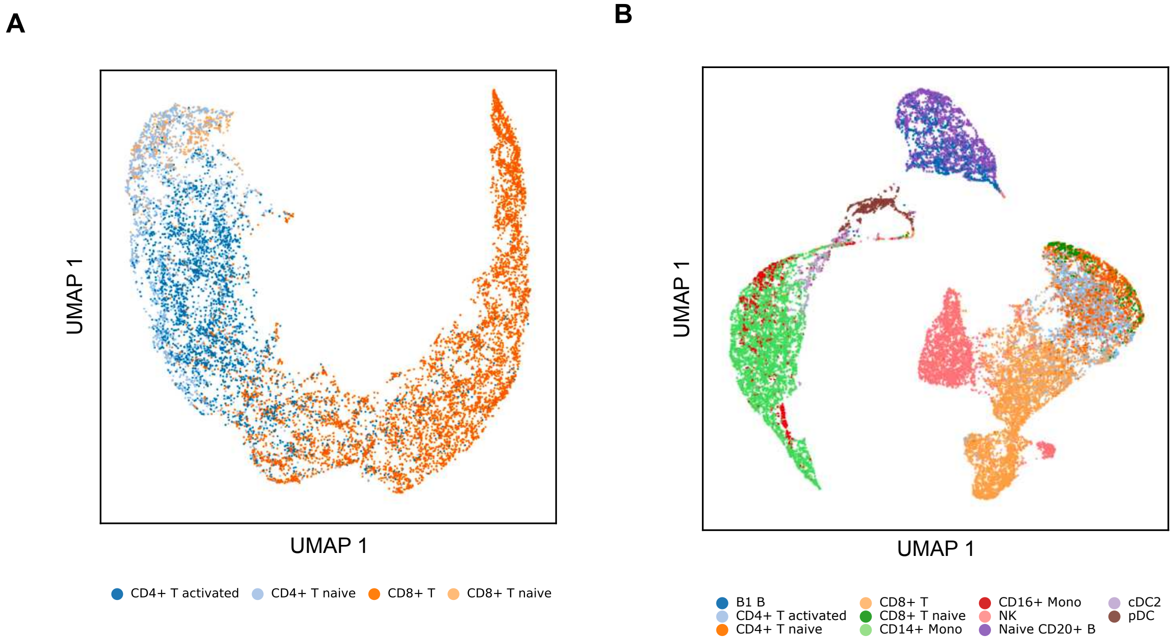

Supplementary Figure S5. scEmbed produces visually distinct clusters of the subsetted Luecken2021 dataset. A. *UMAP plot of an scEmbed model trained at 100 epochs for just the T Cells. Looking only at T Cells, the model can visually cluster the different cell types. B. UMAP plot of only PBMC cells from the Luecken2021 dataset.*

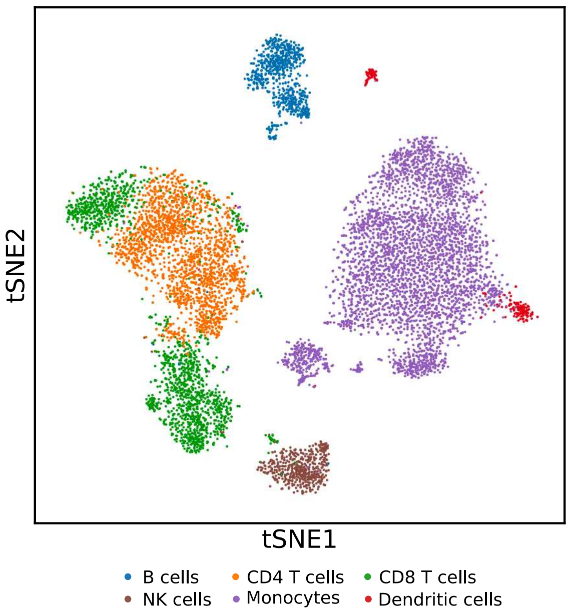

Supplementary Figure S6. Visualization of reference data for Cellcano. *tSNE plot output from cellcano showing clustered data from the given reference dataset.*

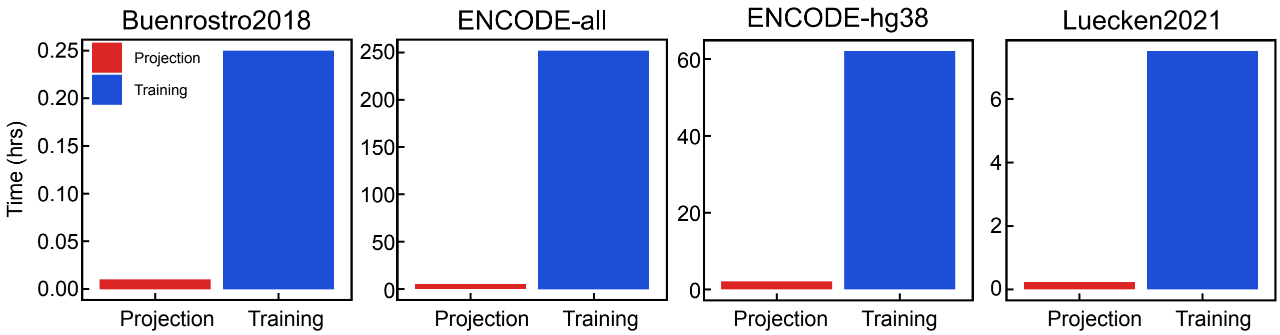

Supplementary Figure *S7. Number of hours required to perform training and projection for various datasets used in this study. Projection of new data into a pre-trained model takes very little time compared to training a model from scratch.*
